# Objective quantification of nanoscale protein distributions

**DOI:** 10.1038/s41598-017-15695-w

**Published:** 2017-11-10

**Authors:** Miklos Szoboszlay, Tekla Kirizs, Zoltan Nusser

**Affiliations:** 1 Laboratory of Cellular Neurophysiology, Institute of Experimental Medicine, Hungarian Academy of Sciences, Budapest, Hungary; 2 János Szentágothai School of Neurosciences, Semmelweis University, Budapest, Hungary

## Abstract

Nanoscale distribution of molecules within small subcellular compartments of neurons critically influences their functional roles. Although, numerous ways of analyzing the spatial arrangement of proteins have been described, a thorough comparison of their effectiveness is missing. Here we present an open source software, GoldExt, with a plethora of measures for quantification of the nanoscale distribution of proteins in subcellular compartments (e.g. synapses) of nerve cells. First, we compared the ability of five different measures to distinguish artificial uniform and clustered patterns from random point patterns. Then, the performance of a set of clustering algorithms was evaluated on simulated datasets with predefined number of clusters. Finally, we applied the best performing methods to experimental data, and analyzed the nanoscale distribution of different pre- and postsynaptic proteins, revealing random, uniform and clustered sub-synaptic distribution patterns. Our results reveal that application of a single measure is sufficient to distinguish between different distributions.

## Introduction

Chemical synapses of the central nervous system substantially differ in their structural, molecular and functional properties (reviewed by^1,2^). Robust diversity is apparent among synapses made by distinct pre- and postsynaptic cell types, which is likely to be the consequence of their distinct molecular makeups. Remarkable functional diversity is also found among synapses made by molecularly and morphologically homogeneous pre- and postsynaptic cells (e.g. hippocampal CA3 pyramidal cells (PCs) on CA1 PCs; refs^3,4^). A plausible explanation for this is that different numbers, densities or distinct nanoscale distributions of the same molecules underlie the functional diversity among these synapses.

Indeed, distinct nanoscale distributions of proteins within the presynaptic active zones (AZs) and postsynaptic densities (PSDs) have been suggested to underlie some forms of functional diversity. Distribution of voltage-gated Ca^2+^ channels (VGCC) governing the Ca^2+^ influx necessary for presynaptic vesicle fusion and their spatial arrangement in relation to the docked synaptic vesicles within the AZ critically affects the probability with which vesicles are released (P_r_; ref.^5^). Analysis of the distribution of the Cav2.1 subunit of VGCCs showed that they are not randomly located within hippocampal^6,7^ and cerebellar^8,9^ synapses and subtle alterations in their sub-AZ locations is sufficient to induce robust changes in synaptic transmission^10^. On the postsynaptic side, AMPA receptor (AMPAR) occupancy declines with distance from the site of vesicle fusion^11^, inferring that the precise distribution of these receptors is of key importance in tuning synaptic strength. A recent study suggested that presynaptic Rim1/2 clusters are aligned to postsynaptic areas densest in GluA2 receptors, endowing increased synaptic strength compared to uniform distribution of postsynaptic receptor^12^. Different AMPAR distribution patterns have also been described in retinogeniculate (RG) and corticogeniculate (CG) synapses formed on relay cells of the dorsal lateral geniculate nucleus^13^. Numerical simulations, however, showed that AMPA receptor-mediated quantal responses were almost identical in RG synapses, displaying homogeneous receptor distributions, and in CG synapses, having similar number of receptors arranged in a clustered manner.

Motivated by these and many other findings, considerable efforts have been dedicated to analyze the spatial arrangement of pre- and postsynaptic molecules and to correlate distinct spatial arrangements with different aspects of synaptic function (e.g. refs^6–8,12–16^). The issue of quantitative analysis of the spatial distribution of point patterns is not unique to neuroscience, a great variety of methods (e.g. several methods based on nearest neighbor (NND) and all-to-all distances; Ripley’s K function and its derivatives; spatial autocorrelation function (ACF); contact distribution) have been developed in various other disciplines (e.g. cell biology^17,18^, ecology, geography, geology, statistical physics; reviewed by^19^). This growing demand led us to develop a Python-based, open source software (GoldExt) with a graphical user interface (GUI), which provides an integrated tool for multi-objective analysis of 2D spatial point patterns. We also provide a detailed comparison of the benefits and effectiveness of the implemented methods and use the best performing ones to experimental data to analyze the spatial distributions of different pre- and postsynaptic proteins.

## Results

### Comparing different measures for distinguishing clustered patterns from random distributions

**Figure 1:**
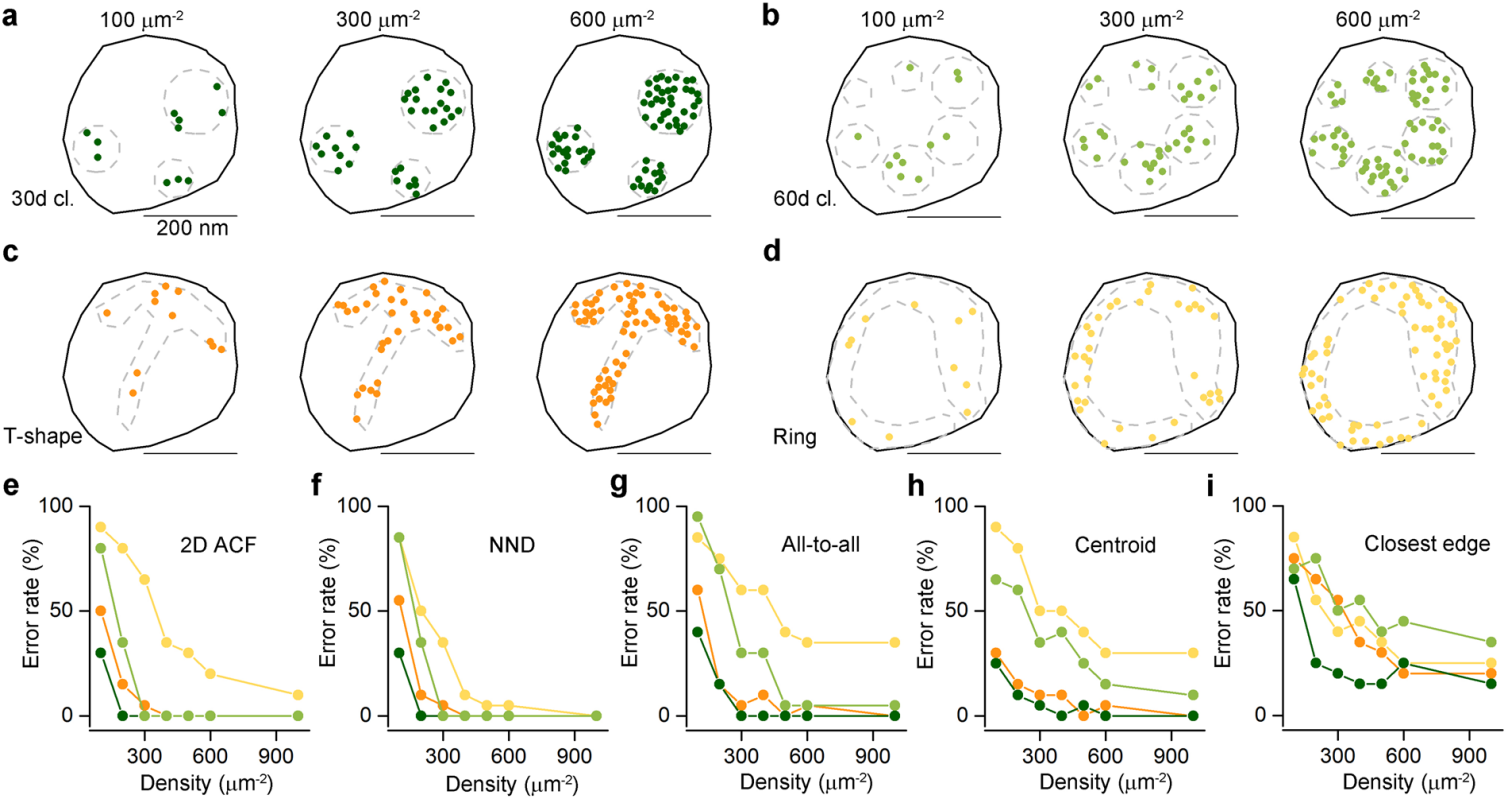
Assessing different measures for discriminating clustered patterns of localization points from random distributions using Monte Carlo simulations. (**a**) Representative multiple-cluster models with localization densities ranging from 100 μm^−2^ to 600 μm^−2^, from left to right. The cluster density is 30 μm^−2^. The dashed grey line delineates the circular areas within which localization points are placed randomly. (**b**) As for **a**, but the density of the clusters is 60 μm^−2^. (**c**) Representative T-shaped models with localization densities ranging from 100 μm^−2^ to 600 μm^−2^, from left to right. (**d**) As for **c**, but for ring-shaped models. (**e**–**i**) Error rates calculated from 20 models, which are individually compared to 200 random distributions. Models with their computed parameter falling <2.5% and >97.5% of that of the random distributions were considered ‘significantly’ different from random. The error rates are plotted as a function of localization densities and are shown for the 5 different measures on the 4 different clustered patterns: ‘2D ACF’: 2D autocorrelation function (**e**); ‘NND’: nearest neighbor distances (**f**), ‘all-to-all’: distance of all localization points to all other localization points (**g**); ‘centroid’: distance of each point from the center of mass of the localization points (**h**); ‘closest edge’: distance of each point from the closest edge of the structure delineating polygon (SDP, **i**).

First, we investigated the efficacy of five different measures in distinguishing clustered patterns of localization points from random distributions. Because many proteins have apparently clustered distributions in the PSD or the AZ^6,12^, we generated clustered distributions of localization points at two different cluster densities (30 μm^−2^: Fig. 1a; and 60 μm^−2^: Fig. 1b) by randomly placing circular areas (randomly selected radii within the range of 25–75 nm) within structure delineating polygons (SDPs) and randomly distributing the localization points within these circular areas (referred to as ‘multiple-cluster’ models). With a mean SDP area of ~0.1 μm^2^, these cluster densities resulted in an average of 3 or 6 clusters per SDPs. Visual inspections of hundreds of electron micrographs of presynaptic AZs immunolabeled for VGCCs or AZ-associated proteins revealed many complex labeling patterns, some of which looked like the letter T or C or a ring. To mimic such patterns, we drew T- (Fig. 1c) and ring-shaped (Fig. 1d) areas within the SDPs and distributed localization points within these areas at different densities. In all four cases, twenty SDPs with somewhat different shapes and sizes were used (mimicking variability of synapses), for which we varied the overall density of localization points (densities are calculated for the whole SDP areas) from 100 μm^−2^ to 600 μm^−2^, with an increment of 100 μm^−2^, and 1000 μm^−2^, covering the range of immunogold densities for synaptic proteins as visualized with SDS-digested freeze-fracture replica immunolabeling (SDS-FRL).

For each of the 560 models (4 patterns, 20 SDPs each, 7 different localization point densities), we performed Monte Carlo simulations by creating 200 corresponding randomizations of localization points and computed five different measures at the level of individual SDPs. Spatial autocorrelation function (*g(r)*; Fig. 1e; ref.^20^) quantifies the probability of finding additional localization points at a certain radius (*r*) from a given localization point. For large rectangular areas, the *g(r)* value of 1 indicates randomly distributed points, (see Methods and refs^12,20^; Fig. S1), whereas values <1 correspond to uniform patterns and values > 1 indicate clustered patterns. As our examined SDPs had highly variable shapes and small sizes, even in the case of random distributions the *g(r)* value slightly deviates from 1 (see Methods). For this reason, we did not use absolute *g(r)* values to determine randomness of a point pattern, but rather compared the mean *g(r)* value of the simulated or experimental data to that of their corresponding randomizations to assess statistical significance. Four distance-based measures were also used: NND (Fig. 1f), all-to-all distances (‘all-to-all’, Fig. 1g), the distance of each point from the center of gravity of the point set (‘centroid’, Fig. 1h) and the distance of each point from the closest edge of the SDP (‘closest edge’, Fig. 1i). The proportion of the 20 SDPs not identified as different from random was then calculated (defined as the error rate; see Methods and Fig. 1). From these five measures, ACF and NND outperformed the others, resulting in an error rate of 0% at all localization point densities above 300 μm^−2^ in case of the multiple-cluster models. Their performance was also remarkably good for the T- and ring-shaped patterns above the localization point density of 400 μm^−2^. These data, taken together, demonstrate that both ACF and NND measures are ideal for distinguishing between random and clustered patterns at the level of individual SDP.

We also performed population-wise comparisons of the simulated datasets with the corresponding random distributions (see Methods). We found highly significant differences (p < 0.001, Kruskal-Wallis test followed by Mann-Whitney *U post hoc* test with Bonferroni adjustment) between the mean *g*(*r*) $(\overline{g(r)})$ and mean NNDs $(\overline{{\rm{NND}}})$ of the simulated data and that of the 200 random distributions even at the lowest tested localization point density of 100 μm^−2^ (Fig. S2 and Table S1). We found that population-wise comparisons are more powerful in detecting differences at low localization point densities than individual SDP level analysis.

### Performance of ACF and NND measures on uniform patterns

**Figure 2:**
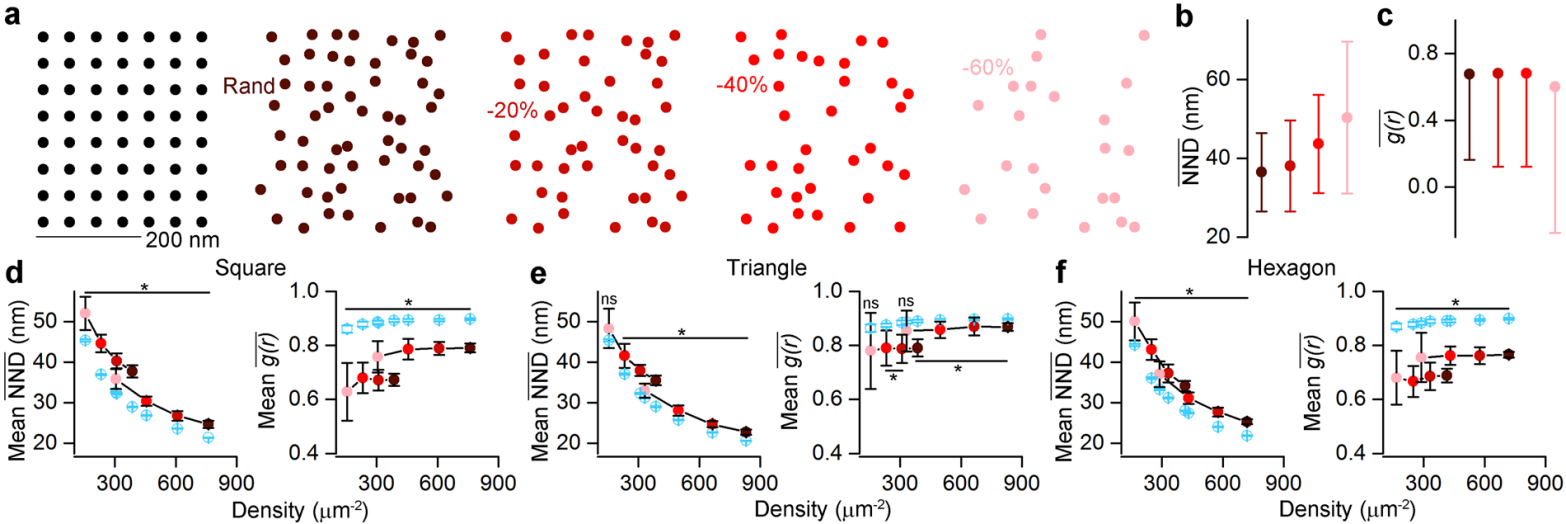
Assessing the effectiveness of the NND and ACF on distinguishing uniform patterns from random distributions. (**a**) Point patterns are generated by randomizing the localization points of a uniform squared grid (two leftmost panels, see Methods). Different labeling efficiencies are modelled by randomly taking away 0%, 20%, 40% and 60% (from left to right) of the localizations points. The 100% ‘labeling efficiency’ has an overall density of 387 μm^−2^. (**b**,**c**)$\,\overline{{\rm{NND}}}$ (**b**) and $\overline{g(r)}$ (**c**) values show weak dependency with the ‘labeling efficiency’. (**d**) Population level comparison of squared uniform patterns (n = 20) and their corresponding randomizations with $\overline{{\rm{NND}}}$ (left) and $\overline{g(r)}$ (right). To cover the previously tested localization point density range, the initial full patterns had a density of either ~400 μm^−2^ (e.g. Rand in **a**) or ~800 μm^−2^. The point pattern distributions originating from the two full patterns are marked by the connected dots (**e**,**f**) Same as in **d**, but for triangular (**e**) and hexagonal (**f**) patterns. The color-coding in **b**–**f** corresponds to the different ‘labeling efficiency’ cases as shown in **a**. Blue symbols represent random distributions. Data are presented as mean ± SD. Wilcoxon signed-rank test was used for statistical comparison. *Indicates Bonferroni corrected p < 0.00625.

ACF and NND seem to be the most powerful measures to differentiate between clustered patterns and random distributions at both single SDP and population levels. Next, we tested how these measures perform when uniform patterns need to be distinguished from random distributions (Fig. 2). We started by simulating one of the most basic uniform patterns, where the localization points are generated by randomizing the location of the nodes of a square grid with a 2D Gaussian having a covariance matrix of [(12^2^,0) (0,12^2^)] (see Methods, Fig. 2a). Motivated by the fact that in immunoreactions, the labeling efficiency is rarely 100%, we tested our approaches on uniform patterns from which 0%, 20%, 40% or 60% of the localizations points were randomly removed (Fig. 2a) and the $\overline{{\rm{NND}}}$ (Fig. 2b) and $\overline{g(r)}$ (Fig. 2c) values were then computed. Our results revealed that the $\overline{{\rm{NND}}}$ and $\overline{g(r)}$ values were not too sensitive to decreasing the ‘labeling efficiency’ (full pattern: $\overline{{\rm{NND}}}$ = 36.5 nm, −60%: $\overline{{\rm{NND}}}$ = 50.3 nm, Fig. 2b; full pattern: $\overline{g(r)}$ = 0.68, −60%: $\overline{g(r)}$ = 0.60, Fig. 2c). Population-level comparison showed that $\overline{{\rm{NND}}}$ values of the uniform patterns are significantly larger than those obtained from random distributions (Fig. 2d) for localization point densities ranging from ~100 μm^−2^ to ~800 μm^−2^, irrespective whether 20%, 40% or 60% of the localization points were removed or not. We also obtained similar findings for triangular (Fig. 2e) and hexagonal (Fig. 2f) patterns. Furthermore, the $\overline{{\rm{NND}}}$ values of the clustered distributions are consistently smaller for the entire tested localization point density range than those of random distributions (Fig. S2a,b). As described previously^12,20^ and shown in our simulations, the $\overline{g(r)}$ values are close to 1 for random patterns and significantly smaller than 1 for uniform patterns (Fig. 2d–f). For clustered patterns, the individual $\overline{g(r)}$ values are substantially higher than 1 (Fig. S2d) and when statistically analyzed at the population level, they are significantly larger than those computed from random distributions (Fig. S2c).

### Clustering of localization point patterns

After analyzing the localization point distribution patterns based on the methods detailed above, an obvious expectation is to investigate whether clusters could be identified or not. Numerous clustering algorithms have been developed, out of which we applied four, in which the number of expected clusters does not need to be pre-defined: DBSCAN (DB; ref.^21^), affinity propagation (AP; ref.^22^), mean shift (MS; ref.^23^) and a recently published algorithm based on Bayesian statistics (Bayesian clustering (BC); ref.^24^). In all of these methods, however, there are user-defined parameters; therefore we started by exploring the parameter space using the above described simulated clustered distributions (Fig. S3). To evaluate the performance of these algorithms on the aforementioned dataset of multiple-cluster models, we calculated the adjusted Rand score (ARS; ref.^25^), which computes similarity measures between two clusterings element-wise. ARS values close to 0 indicate random cluster assignments, whereas an ARS value 1.0 indicates identical cluster assignments. We explored the whole range of localization point densities of the models, and highlighted the data at 400 μm^−2^ (Fig. S3), because that represents the average density of Rim1/2 and Neurexin-1α labeling in our experiments (see details in the next section).

Using the MS method, the ARS has a flat relationship with the minimum number of localization points of the cluster (Fig. S3c) and therefore we selected a minimum number of points of 3 not only for the MS, but also for the DB. In addition to the minimum number of localization points, DB has another user-dependent parameter: ε, which is the maximal distance between two localization points to be considered in the same cluster. The ε vs. ARS curve peaked at 50 nm with a value of 0.94 (indicating almost perfect performance) at the cluster density of 30 μm^−2^ (Fig. S3a), therefore we used an ε of 50 nm throughout the study. AP produced the highest ARS value (0.83) at a ‘preference’ value of −30 (Fig. S3b). For BC, the ARS steeply depends on the parameter ‘extra space’ peaking around 200 nm (Fig. S3d), therefore we added 200 nm extra space to the edges of the SDPs. We also investigated these parameters in our multiple-cluster models with a cluster density of 60 μm^−2^ (Fig. S3e–h), and found that the performance of all algorithms, but DB, substantially dropped.

**Figure 3:**
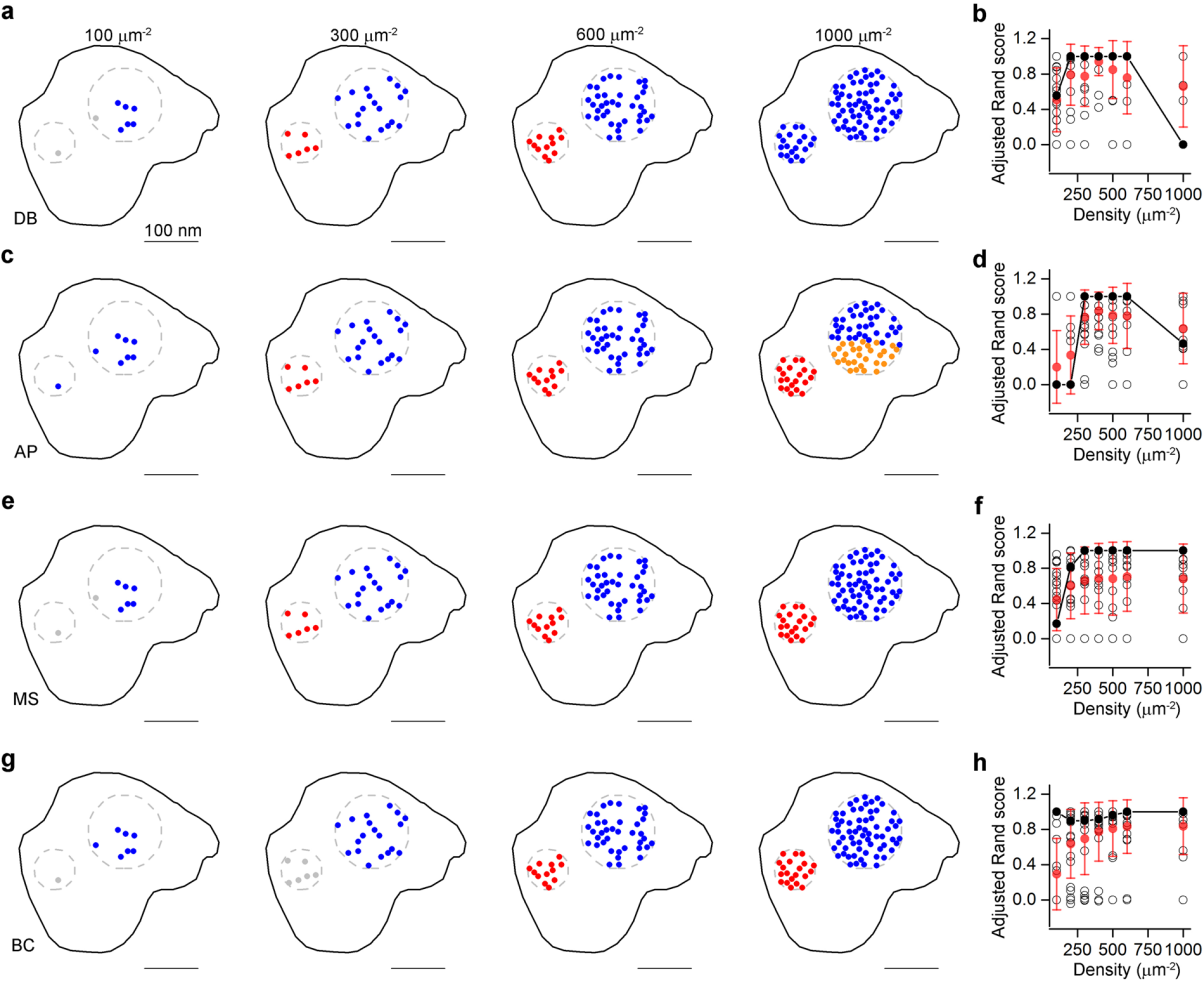
Evaluating the performance of different clustering algorithms. (**a**) Performance of DBSCAN (DB) on a modeled clustered distribution (cluster density: 30 μm^−2^) with different localization point densities. Dashed grey circles demarcate the areas within which the localizations points were randomly distributed. Localization points are color-coded based on their cluster assignment (grey points represent noise). (**b**) Adjusted Rand scores (ARS) computed from 20 different SDPs. (**c–h**) Same as in **a** and **b**, but for the affinity propagation (AP, **c**,**d**), mean shift (MS, **e**, **f**), and Bayesian clustering (BC, **g**,**h**) algorithms. Open black symbols correspond to individual SDPs (n = 20), solid black symbols are the ARS values of the examples shown on the left, red symbols represent mean ± SD.

The four clustering algorithms with user-defined parameters producing highest ARSs were tested on our simulated multiple-cluster distributions and their performances were compared first at a cluster density of 30 μm^−2^ (Fig. 3). Representative example of the same SDP with localization point densities ranging from 100 μm^−2^ to 1000 μm^−2^ is shown from left to right for DB, AP, MS and BC in Fig. 3 with their corresponding ARS values (Fig. 3b,d,f and h for DB, AP, MS and BC, respectively). In case of the representative example, for DB and AP, the performance of the clustering algorithms showed an inverted U shape; the ARS values were low at high and low localization point densities and peaked at localization point densities between 300 and 600 μm^−2^ (Fig. 3, second and third columns). The performance of MS peaked at 300 μm^−2^ and remained high for higher localization point densities and the BC produced consistently high ARS throughout the whole density range tested (Fig. 3f,h). Next, we repeated the same analysis on our artificial multiple-cluster models with a higher cluster density (60 μm^−2^; Fig. S4), with the same user-defined parameters detailed above. Here, there are more clusters for the same SDP areas resulting in less cluster separation. As expected, the performance of most clustering algorithms dropped with the exception of DB and MS at low localization point densities (<300 μm^−2^; Fig. S4a,b). The highest ARS value was obtained with DB at localization point densities of 200 μm^−2^ (~0.8), but this was still substantially lower than the maximum ARS value (0.94) obtained with cluster density of 30 μm^−2^.

### Nanoscale arrangement of different synaptic proteins

**Figure 4:**
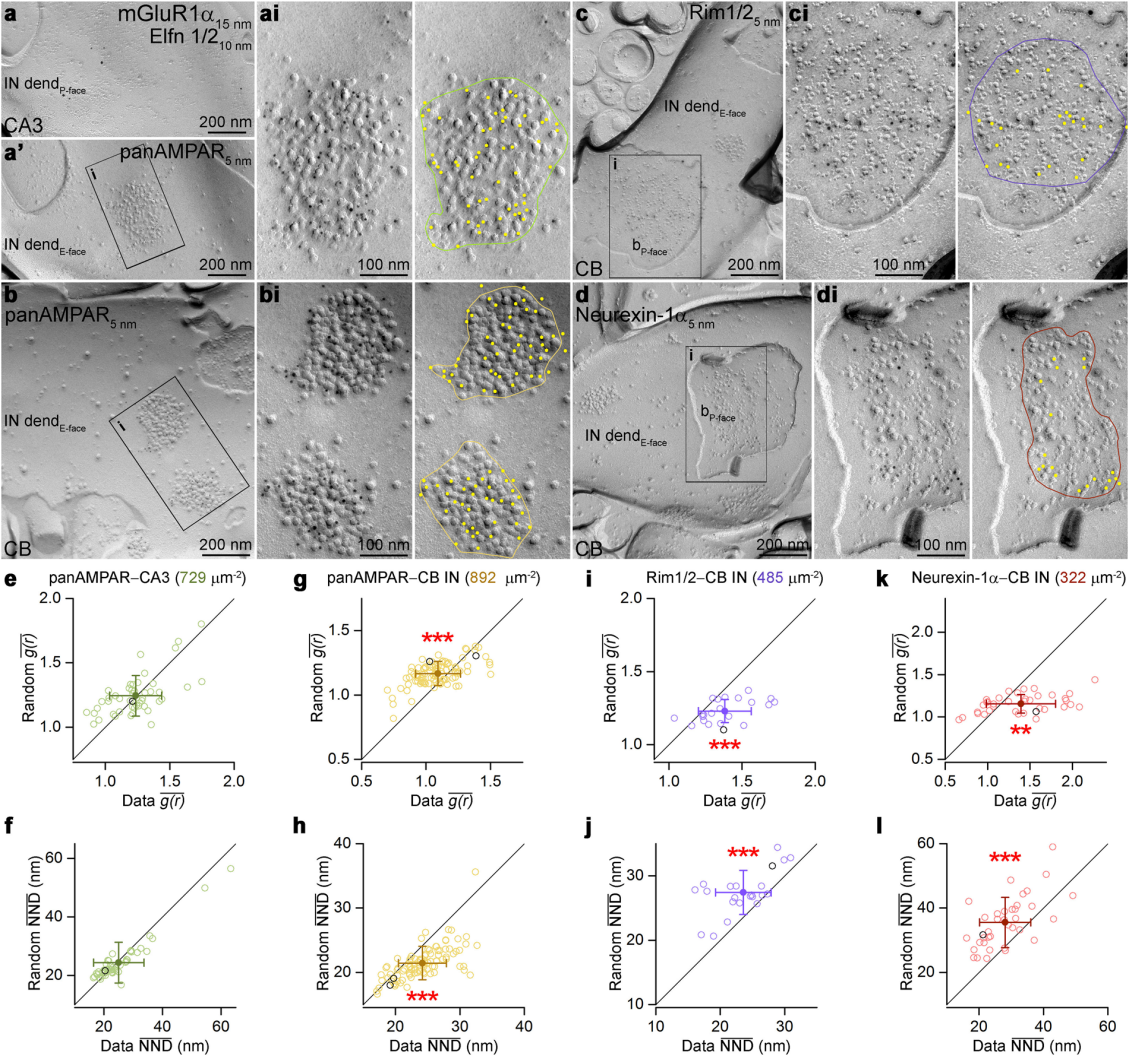
Sub-synaptic distributions of various synaptic proteins. (**a**,**a**’) Mirror replica images of a dendritic plasma membrane segment double labeled for mGluR1α and Elfn1/2 on the protoplasmic-face (P-face), and for AMPARs (using a pan-AMPA antibody) on the complementary exoplasmic-face (E-face) in the stratum oriens of the hippocampal CA3 area. (**ai**) High magnification view of boxed area in **a’** showing a postsynaptic area (PSD) indicated by a dense cluster of intramembrane particles (IMPs) intensely labeled for AMPARs. (**b**) Immunogold labeling for AMPARs on molecular layer interneurons (INs) of the cerebellum (CB). (**bi**) Two PSDs are shown at higher magnification. (**c**,**d**) CB IN dendritic E-face membranes (IN dend) are contacted by bouton P-face membranes that show strong Rim1/2 (**c**) and Neurexin-1α (**d**) immunoreactivity in their active zones (AZs). (**ci**,**di**) High magnification images of the boxed areas in **c** and **d**. On the right panels, the colored lines illustrate the border of the PSDs (**ai**,**bi**) and AZs (**ci**,**di**) with the yellow dots highlighting gold particles. (**e**,**f**) Comparison of the $\overline{g(r)}$ (**e**) and $\overline{{\rm{NND}}}$ (**f**) values obtained from the AMPAR labeling of PSDs of CA3 mGluR1α-positive INs (n = 48 and 49 PSDs, respectively) with random distributions (n = 200 random for each PSD). (**g**–**l**) Same as in **e** and **f**, but for AMPAR (**g**,**h**, n = 95 and 97, respectively), Rim1/2 (**i**,**j**, n = 22) and Neurexin-1α (**k**,**l**, n = 37) labeling in CB IN synapses. Open circles correspond to individual PSDs or AZs; the examples shown in panels a–d are indicated by the black symbols. Filled symbols represent the mean ± SD. Wilcoxon signed-rank test was used for statistical comparison. **Indicates p < 0.01 and ***indicates p < 0.001. b, bouton; dend, dendrite.

We applied the above described methods to experimental data acquired with SDS-FRL to reveal the sub-synaptic distribution of several synaptic proteins. We performed our analysis in the following two well-defined, believed to be homogeneous synapse populations: excitatory CA3 PC synapses on metabotropic glutamate receptor 1α (mGluR1α)-expressing interneurons (INs) in the stratum oriens of the hippocampal CA3 area; and excitatory parallel fiber synapses on molecular layer (ML) INs in the cerebellar cortex (CB). AMPARs are present in most glutamatergic synapses where their sub-synaptic distribution varies depending on the synapse type (e.g. refs^13,15,26–30^). To study the AMPAR distribution in mGluR1α-positive IN dendrites, we performed the ‘mirror replica method’^31,32^: reacting one side of the replicas for mGluR1α (Fig. 4a) and the complementary side for AMPARs (using a pan-AMPA antibody^27^; Fig. 4a’). Gold particles labeling AMPARs were almost exclusively confined to exoplasmic-face (E-face) structures and were highly enriched in PSDs, identified from the high density of intramembrane particles (IMPs; ref.^28^).

To examine the AMPAR distribution within PSDs present on the E-face of mGluR1α immunoreactive dendrites (Fig. 4a’,ai), we calculated the $\overline{g(r)}$ and $\overline{{\rm{NND}}}$ and performed Monte Carlo simulations (n = 200 randomizations for each examined PSD). Both measures indicated that the distribution of AMPAR is not significantly different from random (Fig. 4e,f, p > 0.05, Wilcoxon signed-rank test, n = 48 and 49 PSDs, respectively). In the next set of experiments, we examined the distribution of AMPARs in synapses on ML IN dendrites in the CB (Fig. 4b,bi). Here, the $\overline{g(r)}$ values were significantly smaller than that of their corresponding randomizations ($\overline{g(r)}$: 1.1 vs.1.2) suggesting uniform distribution of AMPARs within these PSDs (Fig. 4g, p < 0.001, Wilcoxon signed-rank test, n = 95 synapses). Consistently, the $\overline{{\rm{NND}}}$ was also significantly larger than that of corresponding random distributions (Fig. 4h, p < 0.001, Wilcoxon signed-rank test, n = 97). Next, we analyzed the sub-synaptic distributions of presynaptic proteins Rim1/2 (Fig. 4c,ci) and Neurexin-1α (Fig. 4d,di) in synapses contacting ML IN dendrites. AZs were delineated based on the underlying high density of IMPs on protoplasmic-face (P-face) membrane fragments. For both Rim1/2 and Neurexin-1α, the $\overline{g(r)}$ was 1.4 and the individual $\overline{g(r)}$ values of synapses were significantly larger than those obtained from the corresponding randomizations (Fig. 4i,k, p < 0.001 and <0.01, n = 22 and n = 37 AZs, respectively, Wilcoxon signed-rank test). As the $\overline{{\rm{NND}}}\,$s in these synapses were also significantly smaller than those of random distributions (Fig. 4j,l, p < 0.001, Wilcoxon signed-rank test, n = 22 and n = 37 AZs, respectively) we concluded that Rim1/2 and Neurexin-1α show clustered distribution patterns in these AZs.

**Figure 5:**
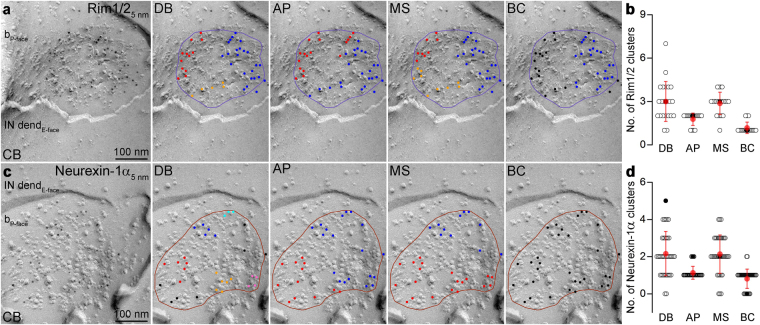
Number of Rim1/2 and Neurexin-1α clusters detected with different clustering algorithms. (**a**) Example for a Rim1/2 decorated IN targeting AZ from the molecular layer of the CB. Panels show the cluster-assignment of gold particles by DB, AP, MS and BC algorithm (different colors represent different clusters, black illustrates noise). (**b**) Number of Rim1/2 clusters detected by the different clustering algorithms. Open circles indicate individual AZs (n = 22 AZs), red symbols represent the mean ± SD. The black filled circles correspond to the AZ shown in **a**. (**c**,**d**) Same as in **a** and **b**, respectively, but for Neurexin-1α (n = 37 individual AZs).

Finally, we performed cluster analysis on the Rim1/2 and Neurexin-1α immunolabeled AZs with the aforementioned four clustering algorithms (Fig. 5) with the previously determined best performing user-defined parameters (Fig. S3). The DB and the MS yielded approximately the same number of clusters (Rim1/2: DB: 3.0 ± 1.4, MS: 2.9 ± 0.8, n = 22 AZs, Fig. 5b; Neurexin-1α: DB: 2.2 ± 1.2, MS: 2.1 ± 1.0, n = 37 AZs, Fig. 5d), whereas the AP and BC algorithms resulted in a somewhat smaller number of clusters for both Rim1/2 (AP: 1.8 ± 0.4, BC: 1.2 ± 0.4) and Neurexin-1α (AP: 1.1 ± 0.3, BC: 0.8 ± 0.5).

## Discussion

We have developed an open source software, GoldExt, with a GUI to provide an integrated analysis tool for quantifying different patterns of 2D protein distributions. Additionally, we tested several well-established clustering algorithms to further analyze clustered point patterns. All of the implemented clustering algorithms have user-dependent parameters, which we fine-tuned on biologically relevant, simulated datasets to achieve the best possible performance before applying them to experimental data. As clustering algorithms detect clusters in essentially any type of point patterns, including random and uniform distributions, we believe that it is necessary first to determine whether the distribution pattern of a given protein is random, uniform or clustered. Our results demonstrate that NND and ACF are the best methods for distinguishing these distribution patterns.

Applying the previously described methods to SDS-FRL data, here we reveal cell-type specific differences in the sub-synaptic distribution patterns for one protein (AMPARs) and fundamentally different patterns for distinct synaptic proteins. First, we have found different AMPAR distribution patterns in INs of the hippocampus and CB. Previously^28^, the AMPAR labeling pattern has been qualitatively described as homogeneous in excitatory PSDs on CB INs. Our data revealed that the NND and ACF of gold particles labeling AMPARs is consistent with uniform patterns in these synapses. In contrast, the AMPAR labeling in the PSDs of hippocampal mGluR1α^+^ IN dendrites was not found to be significantly different from random distributions. To our knowledge, this is the first reported random protein distribution in synapses. The Rim1/2 and Neurexin-1α showed clustered distribution in AZs contacting CB INs, consistent with previous publications showing clustered distribution of Rim1/2, Bassoon and Munc13 in hippocampal AZs^10,12^.

As the size of the investigated structures may differ with orders of magnitude, we tested the robustness of the applied methods for an ion channel subunit (Kv2.1) that is present on the somata and proximal dendrites of CA1 PCs and has been shown to have a clustered distribution^33,34^ (Fig. S5). Although the density of this protein was more than an order of magnitude smaller (~14 μm^−2^) than that of the Rim1/2 and Neurexin-1α (~380 μm^−2^), our measures revealed that the mean NND of gold particles labeling the Kv2.1 subunit is significantly smaller and the mean *g(r)* is significantly larger than that of random distributions (Fig. S5b,c; p < 0.001, Wilcoxon signed-rank test), confirming their clustered arrangement.

## Materials and Methods

### Software

We developed a software (GoldExt) in Python (version 2.7, 64-bit), with which we performed the generation of uniform and clustered patterns; their comparisons to random distributions; and cluster analysis. GoldExt uses the following dependencies: numpy, scipy, matplotlib, scikit-learn^35^, xlsxwriter, openpyxl and PyQt4 (the latter for graphical user interface (GUI), which was drawn using Qt Designer). All the files needed for the generation and running models and data analysis can be found at http://nusserlab.hu/software. GoldExt is developed, tested and ran on 64-bit Windows environment (Windows 10).

### Modelling and data analysis

Four types of models were generated for testing different measures. First, models with different densities of multiple clusters within the structure delineating polygon (SDP) were created. Localization points were randomly distributed within circular areas with radii randomly chosen between 25 and 75 nm. The density of the clusters was either 30 μm^−2^ or 60 μm^−2^. The density of localization points was calculated for the whole SDP area (from 100 μm^−2^ to 600 μm^−2^ with an increment of 100 μm^−2^ and 1000 μm^−2^) and not for the clusters. Two additional types of models were also implemented: ring- and T-shaped models. These shapes were hand-drawn within the SDPs and localization points were randomly placed within them. For the construction of uniform models, we first positioned localization points homogeneously on the nodes of a squared, triangular or hexagonal mesh. Then, we randomized the positions of all the localization points with a 2D Gaussian, having a covariance matrix [(12^2^,0) (0,12^2^)]. The values of the diagonal represent the experimentally constrained variance of the x and y coordinates (in nm, ref.^36^). All models were constructed as a Poisson hardcore process within the above-defined constraints having an inhibition radius of 10 nm (i.e. any two localization points cannot be closer to each other than 10 nm). We categorize the distribution of a point pattern clustered if it is neither random nor uniform.

The following four distance-based measures were implemented to compare experimental data or artificially generated distributions to random distributions: NND, all-to-all distance, distance from the center of gravity of localization points, distance from the nearest edge of the SDP. Once these values were calculated for every localization point, their mean was compared to the mean values of random distributions (200 random distributions per SDP). For individual SDP level comparisons, we considered a localization point distribution different from random, if the mean value of the artificially generated model data was smaller than the 2.5% or larger than the 97.5% of that of the random data (corresponding to 5% significance level). An error rate was calculated as the percentage of SDPs that were not found to be different from their corresponding random distributions.

An additional, more complex measure was also implemented. A spatial autocorrelation function (sometimes referred to as pair autocorrelation function or radial distribution function), *g(r)*, was computed based on^20^. Briefly, the image (I) was binarized (only the pixels which contain localization points become 1, every other pixel has a value of 0) and an image mask (M, which has pixel values of 1 inside the measurement area and is also padded by an equal number of zeros) was created for the different SDPs as the smallest rectangle containing the whole SDP. Since normally the AZ covers only a minor fraction of the whole image, the normalization constant *d*, which is the overall localization density of the image, was calculated within the mask area. Then the *g(r)* function was computed as follows:

1 $$g(r)=\frac{FF{T}^{-1}({|FFT(I)|}^{2})}{{d}^{2}FF{T}^{-1}({|FFT(M)|}^{2})}$$

FFT stands for Fast Fourier Transform and FFT^−1^ for inverse Fast Fourier Transform. Because of the nature of the *g(r)* function, it has a value of 1 in case of random distributions^12,20^. In our case, the area of M is slightly bigger than the SDP area, therefore the localization density *d* becomes a bit lower than the real density inside the SDP, consequently *g(r)* is slightly higher than 1 in case of the random distributions within the SDP. As all SDPs had slightly different shapes, and the ratios of the area of the SDP and the area of the smallest rectangle containing the whole SDP determines the degree of deviation from 1 in every case, we did not use the absolute value of the *g(r)*. Since this deviation is equally present in both the data and its corresponding randomizations, once the *g(r)* functions were computed for the experimental or artificially modelled data and the random distributions, the average values were calculated within the first 80 nm and these averages were compared to each other as detailed above to assess statistical significance. In case of the population-wise comparisons, a simulated dataset was declared clustered or uniform, if its $\overline{g(r)}$ values were significantly larger or smaller, respectively, than that of its corresponding randomizations.

### Clustering

Once a given set of experimental or artificially generated data was found to be statistically different from random, the number of clusters within the data was determined. Clustering was performed with a subset of clustering algorithms implemented in the scikit-learn site-package^35^ of Python. Three algorithms were built into GoldExt, namely DBSCAN (DB, ref.^21^), which is a density-based clustering algorithm, affinity propagation (AP, ref.^22^) and mean shift method (MS, ref.^23^). We also tested our modeled clustered distributions with a Bayesian clustering algorithm (BC; ref.^24^; Fig. 3h and S4). As suggested in the original publication (ref.^24^; Fig. S11a,b), parameters ‘α’ and ‘pbackground’ were set to 20 and 0.5, respectively. We also set an extra 200–200 nm from the edges of the investigated SDPs, which increased the ARS of the algorithm’s output (Fig. S3d,h). For BC, we used the original code provided by the authors, written in R, and ran it in Python using the rpy2 site-package. These algorithms were chosen because the user does not have to determine the number of desired clusters *a priori*.

To evaluate the performance of clustering algorithms on simulated datasets, we computed the adjusted Rand score (ARS; ref.^25^), implemented in the scikit-learn Python site package^35^. Briefly, adjusted Rand score is the Rand score^37^ adjusted for chance. Given *N* points, *X*_1_, *X*_2_, …, *X*_*n*_, and two clusterings of them, *Y* and *Y′*, with arbitrary number of clusters in each clustering and *n*_*ij*_ is the number of points simultaneously in the *i*th cluster of *Y* and the *j*th cluster of *Y′.* The similarity between *Y* and *Y′* is:

2 $$c(Y,\,{Y}^{^{\prime} })=\frac{[(\begin{array}{c}N\\ 2\end{array})-[\frac{1}{2}\{{\sum }_{i}{({\sum }_{j}{n}_{ij})}^{2}+\,{\sum }_{j}{({\sum }_{i}{n}_{ij})}^{2}\,\}-\,{\sum }^{}{\sum }^{}{n}_{ij}^{2}]]}{(\begin{array}{c}N\\ 2\end{array})}$$

The ARS has a value of 0 when the two clusterings have no similarities and 1.0 for identical clustering.

Out of these four clustering algorithms, DB outperformed the others when tested on the artificially generated multiple-cluster models (quantified by the ARS; Fig. 3 and S3, 4) with the following user-dependent parameters: ε = 50 nm, which is the maximum distance between two localization points to be assigned to the same cluster and each cluster has to have at least 3 members (DB); a ‘preference’ value of −30 (AP); and a minimum number of cluster-assigned localization points of 3 (MS).

### SDS-digested freeze-fracture replica-labeling (SDS-FRL)

Two male C57Bl6j mice (P28, P40) and two Wistar rats (P16, P39) were deeply anaesthetized and transcardially perfused with a fixative containing 2% PFA and 0.2% picric acid in 0.1 M PB for 15 minutes in accordance with the Hungarian Act of Animal Care and Experimentation (1998, XXVIII, section 243/1998) and with the ethical guidelines of the Institute of Experimental Medicine Protection of Research Subjects Committee and all methods were performed in accordance with appropriate guidelines and regulations. All experimental protocols were approved by the Protection of Research Subjects Committee of the Institute of Experimental Medicine. 80 μm thick sagittal sections from the cerebellar vermis, horizontal sections from the ventral CA3 and coronal sections from the dorsal CA1 area were cut with a vibratome, cryoprotected in 30% glycerol, frozen with a high-pressure freezing machine (HPM100; Leica Microsystems), fractured with a freeze-fracture machine (BAF060; Leica Microsystems), and processed for SDS-FRL as described previously^34^. Tissue debris was digested from the replicas in a Tris buffered saline (TBS) solution containing 2.5% SDS and 20% sucrose at 80 °C overnight. The replicas were then washed and blocked with 5% BSA in TBS for 1 hour followed by an incubation in a solution of the following primary antibodies: rabbit anti-Elfn1/2 (1:500; Sigma-Aldrich Cat# HPA000781, RRID:AB_1079280), guinea pig anti-mGluR1 (1:1000; Frontier Institute Cat# mGluR1a-GP-Af660, RRID:AB_2531897; ref.^38^), guinea pig anti-panAMPAR (1:100; Frontier Institute Cat# panAMPAR-GP, RRID:AB_2571610; ref.^39^), rabbit anti-panAMPAR (1:1500; Synaptic Systems Cat# 182 403, RRID:AB_10598611), rabbit anti-Rim1/2 (1:1000; Synaptic Systems Cat# 140 203, RRID:AB_887775), rabbit anti-Neurexin-1α (1:100; Frontier Institute Cat# Nrxn-Rb, RRID:AB_2571817), mouse anti-Kv2.1 (1:100; UC Davis/NIH NeuroMab Facility Cat# 75-014 RRID:AB_10673392). Replicas then were washed and incubated in a solution containing the following secondary antibodies: goat anti-mouse IgGs, goat anti-rabbit IgGs and goat anti-guinea pig IgGs coupled to 5, 10 or 15 nm gold particles (1:75 or 1:100; British Biocell). Replicas were rinsed in TBS and distilled water before they were picked up on copper parallel bar grids and examined with a Jeol1011 EM. Antibodies used in this study recognized either intracellular or extracellular epitopes on their target proteins and consequently were visualized by gold particles labeling on the protoplasmic-(P-face) or the exoplasmic-face (E-face), respectively. The nonspecific background labeling was measured on opposite face of the specific labeling of the target proteins.

### Analysis of immunogold distributions in SDPs

To assess the AMPAR in PSDs of mGluR1α-positive IN dendrites, all experiments were performed with the ‘mirror replica method’^31^. Dendrites labeled for mGluR1α on their P-face were chosen in the stratum oriens of the CA3 area, and on the opposing E-face, gold particles labeling AMPAR were counted in PSDs indicated by clusters of IMPs. Gold particles inside the synaptic area and up to 30 nm away from its edge were analyzed. In the CB, AMPAR labeling was examined in PSDs of ML IN dendrites (identified as dendrites without branches, displaying minimum two PSDs and/or contacted by axon terminals). The distribution of Rim1/2 and Neurexin-1α labeling was analyzed in AZs (delineated based on the high density of IMPs), which contacted ML IN dendrites. Only PSDs and AZs fractured in their completeness were considered for this study. To assess the distribution of the Kv2.1 subunit, pictures of P-face membrane segments of putative pyramidal cell somata were taken in the stratum pyramidale of the CA1 region. Then the distribution of gold particles was analyzed on the whole image.

### Statistical tests

Statistical analysis was performed using the Wilcoxon signed-rank test (two paired groups) or Kruskal-Wallis test followed by Mann-Whitney *U* test with Bonferroni adjustment (multiple unpaired groups). Data are presented as mean ± standard deviation (SD).

## Electronic supplementary material

Supplementary Information
