# Supplementary material for: Objective quantification of nanoscale protein distributions
Source: Sci Rep. 2017 Nov 10;7:15240. doi: 10.1038/s41598-017-15695-w (PMC5681686; doi:10.1038/s41598-017-15695-w)
Supplement: Supplementary file 1 — Supplementary Information [file 41598_2017_15695_MOESM1_ESM.pdf]

Objective quantification of nanoscale protein distributions

Miklos Szoboszlay[1,2,3], Tekla Kirizs[1,2,3] and Zoltan Nusser[1*]

[1]Laboratory of Cellular Neurophysiology, Institute of Experimental Medicine, Hungarian Academy of Sciences, Budapest, Hungary,

[2]János Szentágothai School of Neurosciences, Semmelweis University, Budapest, Hungary,

[3]These authors contributed equally to this study

	Density (µm^{-2})	Kruskal–Wallis test p values	Mann-Whitney *U* test p values			
			30d cl.	60d cl.	Ring	T-shape
2D ACF	100	**0.000000002**	0.0000003	0.0012300	0.3104000	0.0000086
	200	**0.000000000**	0.0000001	0.0000001	0.0025600	0.0000001
	300	**0.000000000**	0.0000001	0.0000001	0.0000059	0.0000001
	400	**0.000000000**	0.0000001	0.0000001	0.0000012	0.0000001
	500	**0.000000000**	0.0000001	0.0000001	0.0000021	0.0000001
	600	**0.000000000**	0.0000001	0.0000001	0.0000005	0.0000001
	1000	**0.000000238**	0.0000001	0.0000001	0.0000003	0.0000001
NND	100	**0.000000001**	0.0000001	0.0002469	0.0029200	0.0000097
	200	**0.000000000**	0.0000001	0.0000001	0.0000003	0.0000001
	300	**0.000000062**	0.0000001	0.0000001	0.0000001	0.0000001
	400	**0.000000000**	0.0000001	0.0000001	0.0000001	0.0000001
	500	**0.000000000**	0.0000001	0.0000001	0.0000001	0.0000001
	600	**0.000000000**	0.0000001	0.0000001	0.0000001	0.0000001
	1000	**0.000000000**	0.0000001	0.0000001	0.0000001	0.0000001
All-to-all	100	**0.000000024**	0.0000230	0.0083500	0.0858500	0.0001037
	200	**0.000000002**	0.0000204	0.0123400	0.1404200	0.0000059
	300	**0.000000002**	0.0000170	0.0123400	0.1135500	0.0000067
	400	**0.000000003**	0.0000230	0.0192900	0.2976800	0.0000052
	500	**0.000000002**	0.0000181	0.0105800	0.2732900	0.0000076
	600	**0.000000006**	0.0000204	0.0143600	0.3234800	0.0000097
	1000	**0.000000001**	0.0000141	0.0128200	0.3234800	0.0000045
Centroid	100	**0.000000001**	0.0000008	0.0000525	0.7149800	0.0000001
	200	**0.000000001**	0.0000104	0.0036400	0.2393200	0.0000016
	300	**0.000000001**	0.0000181	0.0071100	0.0909100	0.0000040
	400	**0.000000001**	0.0000230	0.0123400	0.1719300	0.0000035
	500	**0.000000001**	0.0000141	0.0090500	0.1264300	0.0000040
	600	**0.000000003**	0.0000181	0.0055600	0.2393200	0.0000035
	1000	**0.000000001**	0.0000181	0.0105800	0.1719300	0.0000031
Closest edge	100	**0.000052190**	0.0047000	0.0638900	0.0060400	0.1805800
	200	**0.000009368**	0.0039700	0.1332800	0.0065600	0.0810300
	300	**0.000006415**	0.0047000	0.1516500	0.0033400	0.0764300
	400	**0.000020388**	0.0055600	0.1636000	0.0071100	0.0531000
	500	**0.000014837**	0.0051200	0.0909100	0.0105800	0.0638900
	600	**0.000012603**	0.0083500	0.1198600	0.0055600	0.0438800
	1000	**0.000015151**	0.0071100	0.1135500	0.0080200	0.0294400

Table S1. Population level statistical comparisons of simulated data with random distributions.
Kruskal-Wallis test (5 independent groups) was used to test for significant differences between random and clustered distributions in case of the seven densities and five measures. In case of $p < 0.05$ (shown with bold), *post hoc* Mann-Whitney *U* test was applied to compare the four clustered distributions to the random distribution. Red indicates Bonferroni corrected $p < 0.0125$.

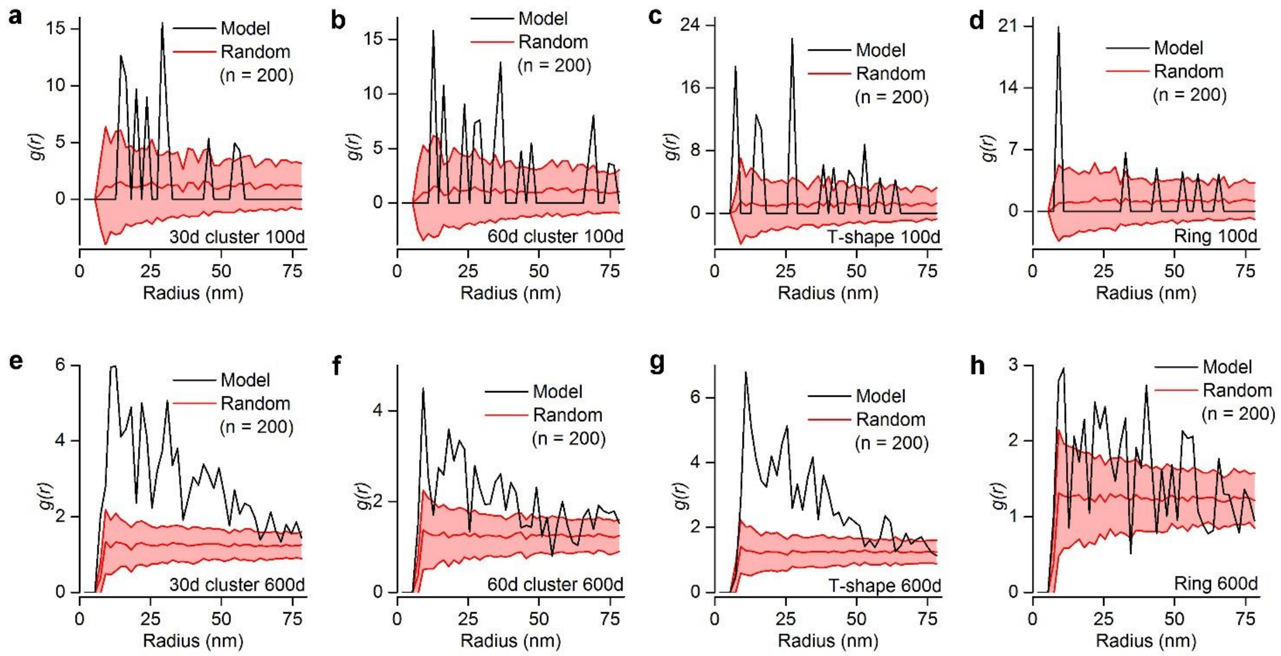

Figure S1: *g(r)* functions of individual models presented in **Figure 1** and that of their corresponding random distributions (n = 200). (**a, b**) Multiple-cluster models with a localization point density of 100 μm^{-2} and cluster densities of 30 μm^{-2} (**a**) and 60 μm^{-2} (**b**). (**c, d**) T-shaped (**c**) and ring-shaped (**d**) models with a localization point density of 100 μm^{-2}. (**e**–**h**) Same as in **a**–**d**, but with a localization point density of 600 μm^{-2}. At the localization point density of 100 μm^{-2} (**a**–**d**) the *g(r)* function often drops back to zero (i.e. the probability of finding another localization point at such distance is zero) with high intermittent peaks. In contrast, the *g(r)* function of models with higher localization densities (e.g. 600 μm^{-2}; **e**–**h**) shows smaller, but more frequent peaks. In all cases, the average *g*(*r*) functions of random distributions are around one. Data of the random distributions are presented as mean ± SD.

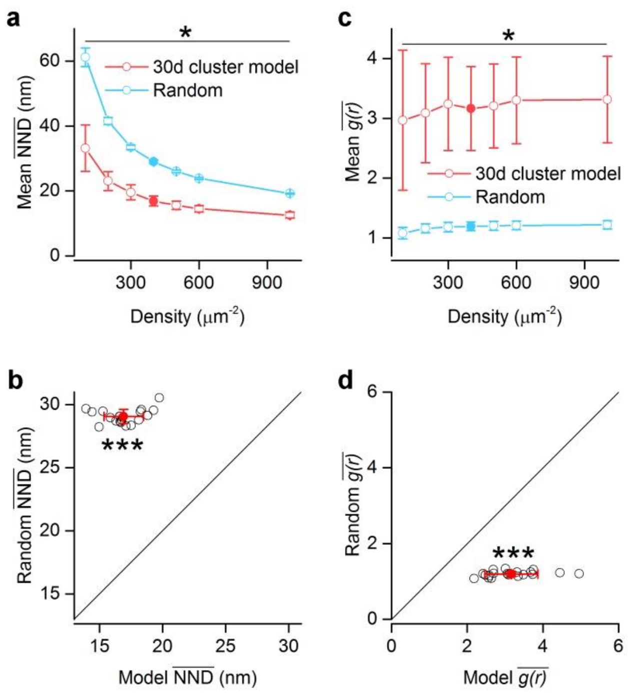

Figure S2: Comparison of multiple-cluster models with their corresponding randomizations. (**a**) Comparing $\overline{\mathrm{NND}}$ values for multiple-cluster models (n = 20 for each localization point density, cluster density: 30 μm^{-2}) with the mean of their corresponding random distributions (n = 200 for each). (**b**) Comparison of individual multiple-cluster models (n = 20, localization density of 400 μm^{-2}, filled circles in **a**) to their corresponding random distributions (from n = 200 randomizations). (**c**, **d**) As in **a** and **b**, respectively, but the comparison was made with $\overline{g(r)}$ function. Data are presented as mean ± SD. Wilcoxon signed-rank test was used for statistical comparison. In **a** and **c** * indicates Bonferroni corrected $p < 0.007$. In **b** and **d** *** indicates $p < 0.001$.

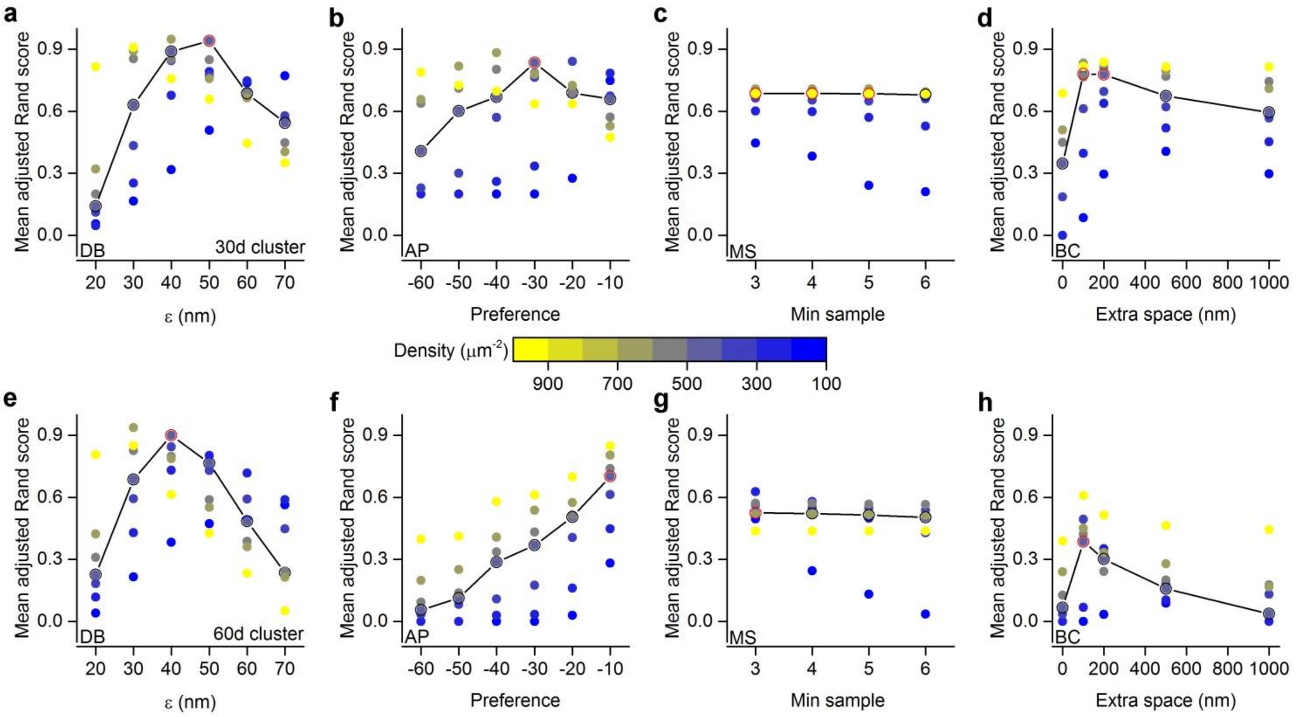

Figure S3: Exploring user-defined variables of different clustering algorithms. (**a**) DBSCAN (DB): mean adjusted Rand scores (ARS) as a function of ε (in nm), which is the maximum distance between two localization points to be assigned to the same cluster. Dots are color-coded based on the densities of the corresponding models (mean of n = 20 SDPs). The line highlights the localization point density of 400 μm^{-2}, which closely matches our experimental data (380 μm^{-2}, n = 59, RIM1/2 and Neurexin-1α). Red open symbol indicates the maximum mean ARS (0.94) at ε = 50 nm. The cluster density is 30 μm^{-2}. (**b**) Mean adjusted Rand scores as a function of the 'preference' value of the affinity propagation (AP) clustering algorithm. The maximum mean ARS (0.83) occurs at 'preference' value of -30 (red open symbol). (**c**) The number of minimum localization points within a single cluster has relatively little effect on the maximum ARS (0.69 (3-5), 0.68 (6), red open symbols) using the mean shift (MS) clustering algorithm. Based on this finding, the same criterion (n = 3 as minimum localization point) was used in case of the DB method as well. (**d**) For Bayesian clustering (BC), multiple user-defined parameters are available, however, we only explored the dependence of the mean ARS on the x-y spatial dimensions of the region of interest (ROI), since the authors of the original publication (Rubin-Delanchy et al., 2015) detailed the dependence of the number of detected clusters on parameters 'α' and 'pbackground' (see figure S11a–b of the original publication), which were set to 20 and 0.5, respectively in our study as suggested by Rubin-Delanchy et al. (2015). In the original paper, the analyzed ROIs were 3000 * 3000 nm. In our preparations, the ROIs were an order of magnitude smaller, so we systematically increased the ROI with additional distances of 0, 100, 200, 500 and 1000 nm outside the SDP borders to explore the dependence of ARS on the size of the ROI. We found that with an extra distance 100 and 200 nm, the mean ARS peaked at 0.78. 200 nm extra space was used for the clustering of experimental data for this algorithm. (**e**–**h**) Same as in **a**–**d**, with a cluster density of 60 μm^{-2}. Note that the performance of the algorithms, excluding DB, drops substantially, when the cluster density in the models was doubled.

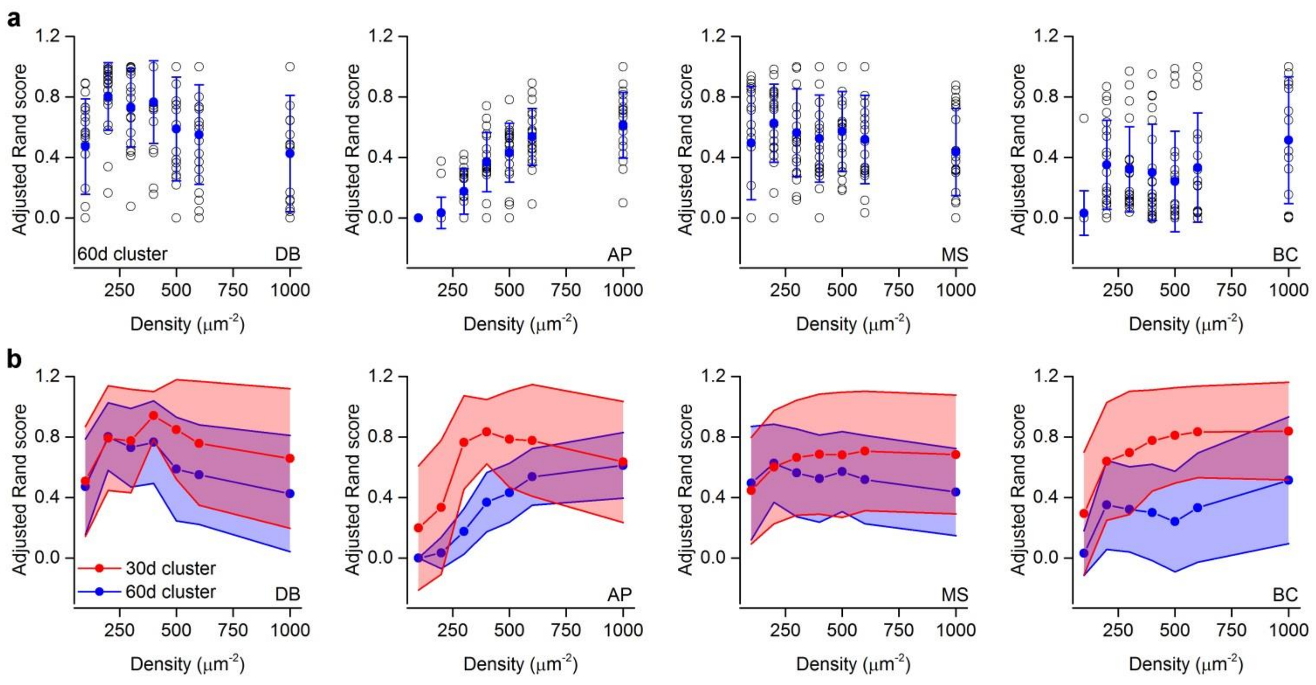

Figure S4: Evaluating the performance of different clustering algorithms. (**a**) Adjusted Rand score (ARS) values of multiple-cluster models (n = 20, open black circles) as a function of localization point density for DB, AP, MS and BC. The cluster density throughout these simulations was 60 μm^{-2}. (**b**) Mean ARS comparison between two cluster densities (30 μm^{-2}: red from Figure 3**b**, **d**, **f** and **h**; 60 μm^{-2}: blue). Note the decrease in clustering performance upon doubling the number of clusters. Data are presented as mean ± SD.

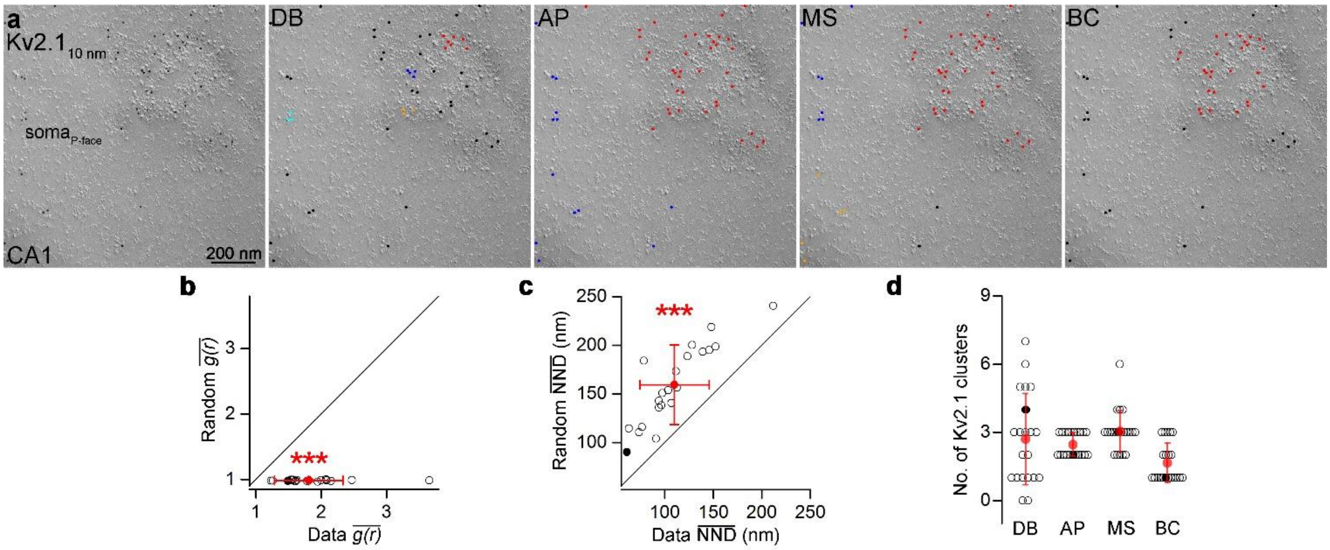

Figure S5: Distribution of Kv2.1 subunit of voltage-gated K$^+$ channels on CA1 pyramidal cells (PCs). (**a**) A P-face plasma membrane fragment of a CA1 PC labeled for the Kv2.1 subunit. Panels on the right show the cluster-assignment of gold particles by DB, AP, MS, and BC (different colors represent different clusters, black illustrates noise). (**b, c**) Comparison of the distribution of Kv2.1 labeling with random distributions (mean of n = 200 random) based on mean $\overline{g(r)}$ (**b**) and $\overline{\text{NND}}$ (**c**). The mean $\overline{g(r)}$ and $\overline{\text{NND}}$ values are significantly larger and smaller, respectively than those of random distributions, indicating that this K$^+$ channel subunit shows clustered distribution. (**d**) Number of Kv2.1 clusters detected by the different clustering algorithms. Open circles correspond to individual images (n = 21 somata), filled circles indicate the example shown in **a**, the red symbols represent mean ± SD. Wilcoxon signed-rank test was used for statistical comparison. *** indicates $p < 0.001$.
